# The *KP1_4563* gene is regulated by the cAMP receptor protein and controls type 3 fimbrial function in *Klebsiella pneumoniae* NTUH-K2044

**DOI:** 10.1371/journal.pone.0180666

**Published:** 2017-07-21

**Authors:** Mei Luo, Shiya Yang, Xuan Li, Pin Liu, Jian Xue, Xipeng Zhou, Kewen Su, Xuan Xu, Ying Qing, Jingfu Qiu, Yingli Li

**Affiliations:** 1 School of Public Health and Management, Chongqing Medical University, Chongqing, China; 2 Dianjiang center for disease control and prevention, Chongqing, China; 3 Zunyi Medical and Pharmaceutical College, Zunyi City, China; Beijing Institute of Microbiology and Epidemiology, CHINA

## Abstract

*Klebsiella pneumoniae* (*K*. *pneumoniae*) is an opportunistic pathogen that can adhere to host cells or extracellular matrix via type 1 and type 3 fimbriae. *KP1_4563* is a gene encoding a hypothetical protein in *K*. *pneumoniae* NTUH-K2044. *KP1_4563* is located between the type 1 and type 3 fimbrial gene clusters and is likely associated with fimbrial function given its putative conserved domains of unknown function (DUF1471). Cyclic AMP receptor protein (CRP) regulates virulence-related gene expression and is a crucial transcriptional regulator in many bacteria. The predicted DNA recognition motif of CRP is present in the *KP1_4563* promoter region. This study aimed to investigate the function of *KP1_4563* in fimbriae and its transcriptional regulation mechanism by CRP. We generated Kp-Δ*4563* mutant and complementation strains. We utilized phenotype and adhesion assays to evaluate the role of *KP1_4563* in fimbriae. We conducted quantitative RT-PCR (qRT-PCR), LacZ fusion, electrophoretic mobility shift, and DNase I footprinting assays to study the transcriptional regulation of *KP1_4563* gene by CRP. We found that *KP1_4563* negatively regulates the function of type 3 fimbriae. Compared with NTUH-K2044, the absence of *KP1_4563* enhanced the ability of Kp-Δ*4563* to adhere to A549 cells. CRP negatively regulates *KP1_4563* by directly binding to its promoter region. *KP1_4563* plays an important role in type 3 fimbrial function. This novel insight will assist in the development of strategies for preventing *K*. *pneumoniae* infection.

## Introduction

*Klebsiella pneumoniae* (*K*. *pneumoniae*), a common hospital-acquired and potentially community-acquired pathogen, causes catheter-associated urinary tract infections, pneumonia, bacteremia, surgical wound infections, pyogenic liver abscesses and bacterial meningitis [1–6]. The ability of bacteria to adhere to host structures plays a major role in the development of infections. In *Enterobacteriaceae*, adhesion is mediated by fimbriae [7–10]. Type 1 and type 3 fimbriae are two commonly expressed and well-characterized fimbriae in *K*. *pneumoniae*. Type 1 fimbriae, which are found in the majority of the *Enterobacteriaceae* family, mediate adhesion to mannose-containing receptors on host cells or in the extracellular matrix [11, 12] and are encoded and regulated by the *fim* gene cluster. Type 1 fimbriae act as virulence factors in urinary tract infections by mediating adhesion to the uroepithelium; these fimbriae also promote the colonization and biofilm formation of *K*. *pneumoniae* on urethral catheters [13–16]. Type 3 fimbriae were first described in the *Klebsiella* species and are common in *Enterobacter*, *Proteus*, *Serratia*, and *Providencia* species [17–21]. Type 3 fimbriae, which are encoded and regulated by the *mrk* gene cluster, adhere to epithelial cells in the respiratory or urinary tracts and to extracellular matrix proteins. Moreover, type 3 fimbriae initiate biofilm formation and are required for biofilm maturation [8, 22–24]. The *K*. *pneumoniae* strain NTUH-K2044 (K1: O1) was first isolated from the blood of a Taiwanese liver abscess patient [25]. In this strain, the type 1 and type 3 fimbrial gene clusters are physically linked. The 4.6-kb DNA fragment between the gene clusters *fim* and *mrk* comprises five open reading frames (ORFs): *KP1_4562* and *KP1_4563*, which are two hypothetical protein encoding genes; *KP1_4564* and *KP1_4565*, which are *pec*M and *pec*S homologues, respectively; and *KP1_4566*, which is a putative high affinity nickel transporter encoding gene [26, 27]. The function of *KP1_456*3 is currently unknown, but the KP1_4563 protein has putative conserved domains of unknown function (DUF1471). DUF1471, also known as PF07338, YhcN, or BhsA/McbA, is a basic feature of sequences in the family of conserved proteins in *Enterobacteriaceae* [28]. Eletsky et.al [29] reported that DUF1471 is involved in the host-pathogen interface. In *Salmonella enterica* Typhimurium, the DUF1471-containing protein YcfR likely influences surface characteristics that mediate surface attachment and cell aggregation [30]. In *Escherichia coli* (*E*. *coli*), YcfR/BhsA has roles that are related to attachment to the surfaces of vegetables [31]. Based on its putative conserved DUF1471 domains, we suspected that *KP1_4563* is associated with adhesion in *K*. *pneumoniae* and influences fimbrial function.

The cyclic AMP receptor protein (CRP), also called catabolite gene activator protein (CAP), is an important global regulator. In the form of the CRP–cAMP complex, CRP enhances the ability of the RNA polymerase holoenzyme to bind and initiate the transcription of specific sets of genes [32–34]. The CRP–cAMP complex globally regulates gene expression in *E*.*coli* by controlling the initiation of transcription of more than 100 operons [35, 36]. CRP is required for carbon metabolism, and regulates the expression of numerous genes that encode bacterial virulence factors, such as flagella, fimbriae, and exotoxins [37–40]. One of our previous studies showed that CRP is an essential virulence regulator: *K*. *pneumoniae* with *crp* knocked out is less virulent in A549 human lung carcinoma cells and in adult female BALB/c mice compared to parental *K*. *pneumoniae* [41]. Several studies have showed that in *K*. *pneumoniae*, the CRP–cAMP complex specifically binds to intergenic regions in *citC-citS* for citrate fermentation and to the promoter proximal region of *allS* for allantoin utilization [41, 42]. The synthetic palindromic DNA recognition motif of CRP is 5′-AAATGTGATCTAGATCACATTT-3′, and it is well characterized in *E*.*coli* [43, 44], The consensus DNA site (underlined above) is the most important site for CRP–DNA complex formation. Sequence analysis identified putative CRP binding sites in the promoter region of *KP1_4563*, which suggests that the CRP–cAMP complex directly regulates the promoter. This hypothesis was confirmed by experimental evidence in this study.

This study aimed to investigate the function and transcriptional regulation mechanism of *KP1_4563*. Thus, we conducted phenotype and adhesion assays to identify the effect of *KP1_4563* on fimbriae function. Subsequently, to study the transcriptional regulation mechanism of *KP1_4563* by CRP, qRT-PCR and LacZ fusion assays were performed to verify the transcription of *KP1_4563*. Furthermore, electrophoretic mobility shift and DNase I footprinting assays were utilized to analyze the specificity of CRP binding to the promoter proximal region of *KP1_4563*.

## Materials and methods

### Bacterial strains, plasmids, primers, and growth conditions

The bacterial strains and plasmids used in this study are listed in Table 1. All the primers used in the present work are listed in Table 2. *K*. *pneumoniae* and *E*. *coli* were grown in Luria–Bertani (LB) medium or LB medium supplemented with antibiotics at the following concentrations: ampicillin (Ap, 100 μg/ml), kanamycin (Km, 50 μg/ml), and chloramphenicol (Cm, 35 μg/ml). To fully express fimbriae, *K*. *pneumoniae* strains were statically cultivated in modified Minka medium for 48 h at 37°C. Continuous cultivation was conducted for three generations at 1:1000 dilution in the same medium [45].

**Table 1:** Bacterial strains and plasmids used in this study.

Strains or plasmids	Genotype or description	Reference or source
*K*. *pneumoniae*		
K2044	K1 serotype	[25]
Kp-Δ*crp*	K2044 with deletion of *crp*	This study
Kp-Δ*4563*	K2044 with deletion of *KP1_4563*	This study
Kpc-Δ*4563*	Kpc-Δ*4563* complemented with *KP1_4563*	This study
CCW01	K2044 Δ*lacZ* strain	[54]
CCW01Δ*crp*	CCW01 with deletion of *crp*	This study
CCW01/placZ15-p*4563*	CCW01 complemented with *KP1_4563*	This study
CCW01Δcrp/placZ15-p*4563*	CCW01Δ*crp* complemented with *KP1_4563*	This study
*E*. *coli*		
DH5α	Cloning host	[66]
BL21	Express the CRP protein	[67]
Plasmids		
pKO3-Km	Km^r^, suicide vector	[68]
pKO3-Km-p*4563*	Km^r^, suicide vector for *KP1_4563* deletion	This study
pBAD33	Cm^r^, cloning vector	Laboratory stock
pBAD33-p*4563*	Cm^r^, cloning vector containing *KP1_4563*	This study
placZ15	Cm^r^, promoter selection vector, lacZ+	[54]
placZ15-p*4563*	Cm^r^, *KP1_4563* promoter fused with lacZ reporter	This study

**Table 2:** Oligonucleotide primers used in this study.

Primers	Sequence (5'-3')
Gene deletions	
*KP1_4563*-A	ATAAGAAT**GCGGCCGC**GGCGATGCTGATTTATGC
*KP1_4563*-B	CCCTCTGCAACCATTCGCGTTTGCTTTCGATGGACTT
*KP1_4563*-C	AAGTCCATCGAAAGCAAACGCGAATGGTTGCAGAGGG
*KP1_4563*-D	ATAAGAAT**GCGGCCGC**TCGGGGCGATCAGTATGG
Complementation of mutant	
*KP1_4563*-HB-KpnI-F	CGG**GGTACC**AGGAGG*AATTCACC*ATGCTTTCCACCATAAAA
*KP1_4563*-HB-SalI-R	ACGC**GTCGAC**TTATTTAGACAGCTCGGC
qRT-PCR	
*KP1_4563*-RT-F	CGGTATGCTCTCCCTGGTC
*KP1_4563*-RT-R	TATTTAGACAGCTCGGCGGTC
LacZ fusion	
*KP1_4563*-LacZ-F	CGC**GGATCC**CGATGCTGATTTATGCCAC
*KP1_4563*-LacZ-R	GGA**AGATCT**ATACCGGCAGCTGCGAGTAA
Protein production	
*KP1_5071*-CRP-P-F	GCG**GGATCC**ATGGTGCTTGGCAAACCG
*KP1_5071*-CRP-P-R	GCG**AAGCTT**TTAACGGGTGCCGTAGACG
EMSA	
*KP1_4563*-EMSA-F	CGATGCTGATTTATGCCAC
*KP1_4563*-EMSA-R	ATACCGGCAGCTGCGAGTAA
*KP1_16S* -EMSA-F	CGGTCTGTCAAGTCGGATGTG
*KP1_16S* -EMSA-R	CGGAAGCCACGCCTCAAG
DNase I footprinting	
*KP1_4563*-FP-F	ATGTGATACCCCCTTTCAGAAG
*KP1_4563*-FP-R	ATACCGGCAGCTGCGAGTAA

Amplification of the *KP1_4563* coding region together with AGGAGG, which is a ribosome binding site (underlined) consensus sequence, and AATTCACC (italic), a spacer. Bold letters indicate the respective restriction enzyme site in the primer.

### Construction of gene deletion and complementation strains

Mutant Kp-Δ*4563* was constructed via a previously described unmarked deletion method [46, 47]. In brief, the upstream and downstream flanking DNA fragments of *KP1_4563* were amplified. The two flanking fragments were fused by PCR and then cloned into the temperature-sensitive suicide vector pKO3-Km. The recombinant plasmid was introduced into K2044 by electroporation. Integration (at 30°C) and excision (at 43°C) of the plasmid generated Kp-Δ*4563*, as confirmed by PCR and DNA sequencing.

To construct the complementation strain, the DNA region that contained the intact *KP1_4563* gene was amplified via PCR. The DNA fragment was then cloned into the pBAD33 plasmid. Then, the recombinant plasmid was introduced into Kp-Δ*4563* via electroporation. The Kpc-Δ*4563* complementation strain was selected on LB agar plates supplemented with chloramphenicol and verified via PCR.

### Hemagglutination assays

The expression of type 3 fimbriae was examined via mannose-resistant hemagglutination (MRHA) assays as previously described [17, 48]. Briefly, strains were statically cultivated in modified Minka medium. The third generations of the strains were harvested, washed once with phosphate-buffered saline (PBS), and resuspended at a concentration 10^10^CFU/ml. Then, 2.5% of fresh human erythrocytes were treated with an equal volume of a 0.003% (wt/vol) tannic acid (Sigma) solution in saline for 10 min at 37°C and were washed twice with PBS. Tanned erythrocytes were mixed with equal volumes of a series of 2-fold dilutions of the bacterial suspension with or without 0.25% mannose (Sigma) in 96-well, U-bottomed microtiter plates. The plates were gently agitated at room temperature for 1 min. Then, the minimum bacterial density (CFU/ml) required to agglutinate erythrocytes was measured. The expression of type 1 fimbriae was specifically detected via the mannose-sensitive agglutination of guinea pig red blood cells (RBCs) assays [49]. As described above, 2.5% guinea pig RBCs were mixed with a series of 2-fold dilutions of bacterial suspension with or without 0.25% mannose in 96-well, U-bottomed microtiter plates. Two controls were included in this experiment: *E*. *coli* DH5α (type 1 fimbriae expression) and PBS (negative control). The plates were gently agitated at room temperature for 1 min. Then, the minimum bacterial density (CFU/ml) required to agglutinate erythrocytes was measured. The experiment was repeated at least thrice.

### Mannan-binding assay

The quantity of fimbriae from the K2044, Kp-Δ*4563*, and Kpc-Δ*4563* strains was examined by mannan-binding assay as previously described [50]. Mannan (Sigma) derived from *Saccharomyces cerevisiae* was dissolved in 0.02 M bicarbonate buffer. Then, 100 μl of 20 μg/ml mannan was statically incubated in 96-well, flat-bottomed cell culture plates at 37°C for 1 h. The wells were washed thrice with sterile PBS (pH 7.4) and quenched with 0.1% bovine serum albumin (BSA) at 37°C for 15 min. The WT, Kp-Δ*4563* and Kpc-Δ*4563* strains were statically cultivated in modified Minka medium under the same conditions described above. The third generations of strains were adjusted to OD_540_ = 2.0. Then, 100 μl of bacterial solution was added to the wells. The strains were then statically incubated at 37°C for 45 min. After incubation, unattached bacteria were removed by washing the wells thrice with sterile PBS. A total of 150 μl of modified Minka medium was added to per well. The strains were then incubated at 37°C for 4 h with shaking at 200 rpm. Finally, the density of bound bacteria in each well was determined by measuring the OD_415_ with an Absorbance Microplate Reader (BioTek, USA). Each strain was assayed with six technical replicates in each experiment, and at least three biological replicates were performed.

### Bacterial adhesion assays

The adhesion ability of the K2044, Kp-Δ*4563*, and Kpc-Δ*4563* strains was examined by adhesion assays as previously described [51]. Briefly, monolayers of A549 human lung epithelial cell lines (8×10^5^) were infected at a multiplicity of infection (MOI) of 100 in 24-well, flat-bottomed cell culture plates, followed by incubation at 37 °C for 4 h. After incubation, the wells were washed thrice with PBS to remove unattached bacteria. Adherent bacteria were released by the addition of 1 ml of 0.1% Triton per well and were then quantified by plating appropriate dilutions on LB agar plates. Adhesion was expressed as the number of CFU that adhered to the A549 cells. The results were presented as the mean of at least three technical repeat wells in each experiment, and at least three biological replicates were performed.

### Quantitative RT-PCR (qRT-PCR)

K2044 and Kp-Δ*crp* strains were statically cultivated in modified Minka medium at 37°C for 48 h. The third generations of the strains were diluted 1:1000 in 15 ml of fresh medium and grown until OD_600_ = 1.0. The bacteria were pelleted for RNA extraction. RNA was extracted, and the residual DNA was eliminated with an RNAprep Pure Cell/Bacteria Kit (TIANGEN) in accordance with the manufacturer’s instructions. RNA quality was determined via 1.2% agarose gel electrophoresis, and RNA quantity was determined with a NanoDrop 2000 UV-Vis Spectrophotometer (Thermo Scientific). Equal quantities of RNA were converted to cDNA using the random hexamer primer from a RevertAid First Strand cDNA Synthesis Kit (Thermo Scientific). Real-time PCR was performed with a SYBR Premix Ex Taq II Kit (Takara) and LightCycler System. Data were normalized with 16S rRNA as the endogenous reference. The relative expression ratio of a target gene was calculated using a previously described method [52]. Every sample was tested in triplicate in each experiment. Each experiment was repeated at least thrice.

### LacZ fusion and β-galactosidase assay

The promoter region of *KP1_4563* was amplified from K2044 using the primers listed in Table 2. The DNA fragments were cloned into the *Bam*HI and *Bgl*II sites of the placZ15 plasmid [53]. The recombinant placZ15-p*4563* plasmid was verified via PCR, and then introduced into *K*. *pneumoniae* NTUH-K2044Δ*lacZ* strain CCW01[54] and the deletion mutant CCW01Δ*crp*. The bacteria carrying different plasmids were statically cultivated in modified Minka medium. β-galactosidase activity in cellular extracts was tested by using the β-Galactosidase Enzyme Assay System (Promega). Promoter activity was expressed as Miller units. Every sample was tested in triplicate in each experiment, and was at least three biological replicates were performed.

### Electrophoresis mobility shift assay (EMSA)

The His–CRP protein was purified as previously described [41, 55]. For the EMSA [56, 57], the putative promoter region fragments of *KP1_4563* were labeled at the 5′ end with [γ-^32^P] ATP and T4 polynucleotide kinase. In the final 10 μl of the reaction volume, the labeled DNA fragment (1000 to 2000 cpm/μl) was incubated with increasing amounts of purified His–CRP protein and 20 mM cAMP at room temperature for 20 min in 5× binding buffer. Three controls were included in the EMSA experiment: 1) cold probe (the same promoter-proximal DNA region unlabeled) as a specific DNA competitor, 2) negative probe (the unlabeled coding region of the 16S rRNA gene) as a non-specific DNA competitor, and 3) unrelated protein as a non-specific protein competitor. After incubation, the samples were analyzed by electrophoresis on 4% native polyacrylamide gels in 0.5× TBE buffer at 80–120 voltage and 4°C. Radioactivity was detected via autoradiography after exposure to Kodak film at -70°C.

### DNase I footprinting

For the DNase I footprinting [56, 57], the sense or antisense primer of the putative promoter region of the *KP1_4563* gene was labeled with [γ-^32^P] ATP and T4 polynucleotide kinase. The putative promoter region of *KP1_4563* was amplified by PCR with ^32^P-labeled primers. In the final 10 μl of the reaction volume, the purified PCR products (15,000 to 20,000 cpm/μl) were incubated with increasing amounts of purified His–CRP protein with 20 mM cAMP in 5× binding EMSA buffer. The reaction volume was incubated at room temperature for 30 min. Optimized DNase I (Promega) was then added to the reaction mixture, and it was incubated at room temperature for 30–70s. The reaction was quenched by adding 9 μl of stop solution (200 mM NaCl, 30 mM EDTA, and 1% SDS). Then, the mixture was incubated at room temperature for 1 min. The digested DNA was extracted with phenol/chloroform and analyzed on 6% polyacrylamide gels with 8 M urea. Radioactivity was detected as above. Footprints were identified by comparison with sequence ladders.

### Nucleotide sequence accession number

All sequences obtained in this study were deposited in GenBank under the accession number NC_012731.

### Statistical analyses

Statistical analyses were conducted with SPSS 22.0 software. *P*<0.05 was considered statistically significant.

## Results

### The *KP1_4563* gene negatively regulates the function of type 3 fimbriae

We successfully generated Kp-Δ*4563* and Kpc-Δ*4563* strains. We then utilized phenotype and adhesion assays to investigate the role of *KP1_4563* in fimbriae.

**Fig 1:**
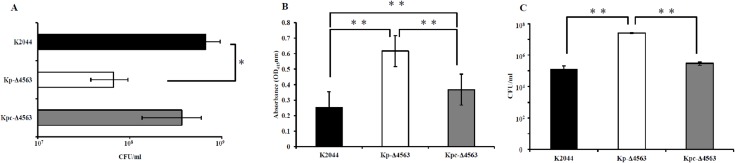
Phenotype and adhesion assays of *KP1_4563*. (A) Hemagglutination assays. Mannose-resistant hemagglutination (MRHA) assays were performed with human erythrocytes. The results are expressed as the minimum bacterial density (CFU/ml) required to cause a visible agglutination reaction. Values represent the mean of three independent experiments, and the error bars represent standard deviation. *P* values were calculated by one-way ANOVA and Tukey HSD post hoc comparisons. (B) Mannan-binding assay. Mean values and standard deviation of six technical replicates are showed. *P* values were calculated by one-way ANOVA and LSD post hoc comparisons. (C) Bacterial adhesion assays. Data are the means of measurements made in technical triplicates. Error bars represent the standard deviation. *P* values were calculated by one-way ANOVA and LSD post hoc comparisons. Significant differences are indicated by * for *P*<0.05 or ** for *P*<0.01.

To address the influence of *KP1_4563* on type 3 fimbrial function, we performed MRHA, a sensitive functional assay for type 3 fimbriae. The minimum bacterial density (CFU/ml) that was required to agglutinate erythrocytes is showed in Fig 1A. Deleting *KP1_4563* from K2044 increased the MRHA activity. The minimum bacterial density of Kp-Δ*4563* required to agglutinate erythrocytes was 6.67×10^7^ CFU/ml, which was approximately 10-fold lower than that of K2044 (6.67×10^8^CFU/ml, *P*<0.05). The MRHA activity weakened when Kp-Δ*4563* was complemented with pBAD33-p*4563* plasmid, and the minimum bacterial density of Kpc-Δ*4563* required to agglutinate erythrocytes was 3.67×10^8^ CFU/ml. Two controls were included in this experiment, *E*. *coli* DH5α (type 1 fimbriae expression) and PBS (negative control), which both failed to mediate visible agglutination.

The effect of *KP1_4563* on the function of type 1 fimbriae was determined via the mannose-sensitive agglutination assays of guinea pig RBCs. The highest bacterial densities (>1×10^10^CFU/ml, data not showed) of K2044, Kp-Δ*4563*, and Kpc-Δ*4563* examined all failed to induce the visible mannose-sensitive agglutination of guinea pig RBCs. Two controls were included in this experiment, *E*. *coli* DH5α, which mediates visible mannose-sensitive agglutination, and PBS, which does not induce agglutination. These results indicated that *KP1_4563* is irrelevant to the function of type 1 fimbriae.

Type 1 and type 3 fimbriae both bind to yeast surfaces [49]. A binding assay with mannan derived from *Saccharomyces cerevisiae* (the receptor compound) was performed to investigate the function of fimbriae. As showed in Fig 1B, compared with K2044, Kp-Δ*4563* bound to mannan more strongly. Binding was reduced when Kp-Δ*4563* was complemented with the expression-complementary pBAD33-p*4563* plasmid. These results indicated that *KP1_4563* likely negatively regulates the function of type 3 fimbriae in an unknown manner.

### Role of *KP1_4563* gene in bacterial adhesion

The ability of K2044, Kp-Δ*4563*, and Kpc-Δ*4563* to adhere to A549 human lung cancer cells were analyzed (Fig 1C). The absence of *KP1_4563* dramatically enhanced the adhesion of Kp-Δ*4563* to A549 cells compared with that of K2044. Adhesion was weakened when Kp-Δ*4563* was complemented with the expression-complementary pBAD33-p*4563* plasmid.

### CRP negative regulates *KP1_4563*

**Fig 2:**
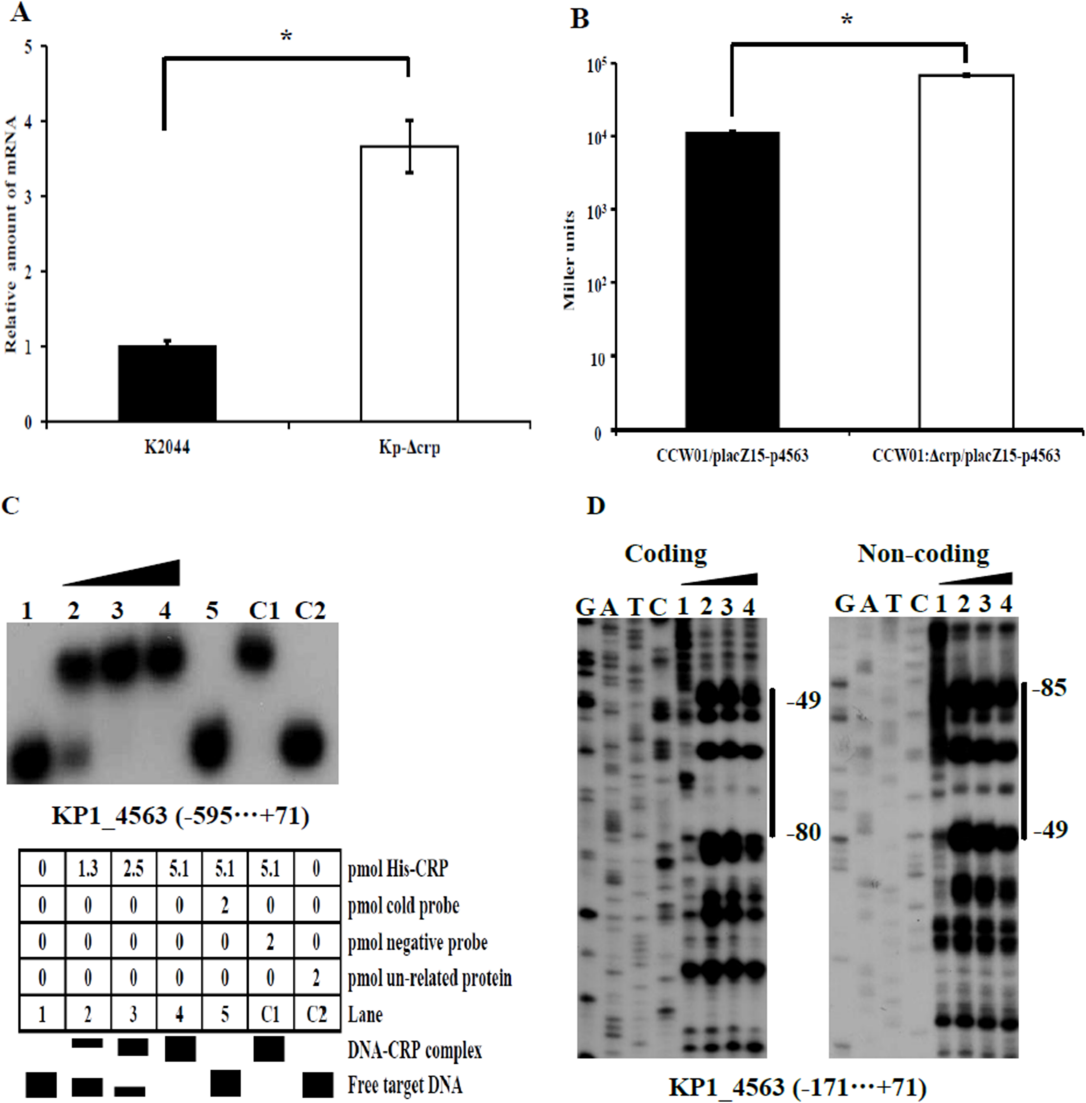
Transcriptional regulation of *KP1_4563* by CRP. (A) Quantitative RT-PCR (qRT-PCR). Transcriptional expression of *KP1_4563* in WT and Kp-Δ*crp*. The results are expressed as the percentage of WT expression. Data are presented as the mean of at least three technical replicates (mean ± standard deviation). Statistical significance was analyzed by independent samples *t*- test. Significant difference is indicated by * for *P*<0.05. (B) LacZ fusion assay. The putative promoter region of *KP1_4563* was cloned into the *lacZ* transcriptional fusion placZ15 plasmid and then introduced into CCW01 or CCW01Δ*crp* to determine promoter activity. The results are expressed as β-galactosidase activity (Miller units) in the cellular extracts. Statistical significance was analyzed by independent samples *t*-test. Significant difference is indicated by * for *P*<0.05. (C) EMSA. The radioactively labeled putative promoter region of *KP1_4563* was incubated with increasing amounts of purified His–CRP protein with cAMP and was then subjected to 4% (w/v) native polyacrylamide gels electrophoresis. The interaction between His–CRP and the promoter region of *KP1_4563* formed a DNA–CRP complex, which produced a retarded DNA band with decreased mobility. (D) DNase I footprinting. A labeled coding or non-coding DNA fragment was incubated with increasing amounts of His–CRP (lanes 1, 2, 3, and 4 represent 0, 8.5, 16.9, and 25.4 pmol of purified His–CRP protein, respectively) with cAMP and was then subjected to 8 M urea-6% (w/v) polyacrylamide gels electrophoresis. The footprint region is indicated by vertical bars with positions, and the negative numbers indicate the nucleotide positions upstream of the *KP1_4563* gene start codon ATG where the A in the ATG start codon refers to position 1.

To investigate the regulation of *KP1_4563* by CRP, qRT-PCR, LacZ fusion, electrophoretic mobility shift, and DNase I footprinting assays were performed. We monitored the expression of *KP1_4563* in K2044 and the Kp-Δ*crp* mutant via qRT-PCR (Fig 2A). Compared with K2044, the expression levels of *KP1_4563* increased 3.7-fold (*P*<0.05) in the Kp-Δ*crp* mutant. The results of the LacZ fusion assays showed that the activity of β-galactosidase in CCW01Δ*crp*/placZ15-p*4563* (6.8×10^4^ Miller units) increased approximately 6-fold relative to that of CCW01/placZ15-p*4563* (1.14×10^4^ Miller units, *P*<0.05, Fig 2B). The results of qRT-PCR and the LacZ fusion assays revealed that CRP negatively regulates *KP1_4563*. EMSA was performed to determine the specificity of CRP binding to the upstream region of the translation start site of *KP1_4563*. As showed in Fig 2C, purified His–CRP protein bound to the upstream region of *KP1_4563* DNA fragments in a dose-dependent manner. Positive EMSA results were observed for *KP1_4563*, which demonstrated that the CRP–cAMP complex directly bound to the *KP1_4563* promoter. The DNase I footprinting showed that His–CRP protected a single DNA region upstream of the *KP1_4563* gene in a dose-dependent manner (Fig 2D). The binding site ranged from 49 bp to 85 bp upstream of the *KP1_4563* start codon ATG, where the A in the ATG start codon refers to position 1. Therefore, CRP likely negatively regulates the transcription of *KP1_4563* by directly bindng to the promoter region.

## Discussion

The region between the *fim* and *mrk* fimbrial gene clusters is highly conserved in different *K*. *pneumoniae* isolates. Sequence analysis of the fimbrial region from *K*. *pneumoniae* C132-98, C747, and C4712 has revealed the presence of homologues of the five ORFs described in *K*. *pneumoniae* C3091 [26]. *KP1_4563* is a hypothetical protein-encoding gene in the fimbrial region. The KP1_4563 protein has putative conserved domains of unknown function (DUF1471). This study describes, for the first time, the role of *KP1_4563* in type 3 fimbrial function and elucidates the transcriptional regulation of *KP1_4563* by CRP. The function of *KP1_4563* reported here will facilitate understanding of the functions of other DUF1471 proteins’ function. Understanding the effect of the *KP1_4563* on type 3 fimbrial function will aid in the development of strategies for preventing *K*. *pneumoniae* infection.

We investigated fimbrial types in K2044, Kp-Δ*4563*, and Kpc-Δ*4563*. The majority of the *K*. *pneumoniae* strains express the type 1 and type 3 fimbriae [58–60]. Type 1 fimbrial expression is specifically detected by the mannose-sensitive agglutination of guinea pig RBCs, whereas type 3 fimbrial expression is detected by the agglutination of tannic acid-treated human erythrocytes in a mannose-resistant manner [17, 49]. This study is the first to describe the effects of the *KP1_4563* gene on fimbriae. Unexpectedly, K2044, Kp-Δ*4563*, and Kpc-Δ*4563* all failed to mediate the visible mannose-sensitive agglutination of guinea pig RBCs at the highest tested bacterial density (>1×10^10^CFU/ml, data not showed). These results indicated that *KP1_4563* does not influence type 1 fimbrial function. The minimum bacterial density of Kp-Δ*4563* required to agglutinate tannic acid-treated human erythrocytes was approximately 10-fold lower than that of K2044. The results of the mannan-binding assay further confirmed the negative regulatory role of *KP1_4563* in type 3 fimbriae.

The ability of bacteria to adhere to host structures plays a major role in the development of infections. Given that type 3 fimbriae can adhere to epithelial cells in the respiratory tract, A549 human lung epithelial cell lines were selected as target cells to identify the effect of *KP1_4563* on adhesion in *K*. *pneumoniae*. As expected, the ability of Kp-Δ*4563* to adhere to A549 cells was dramatically enhanced. The results showed that the absence of *KP1_4563* increased bacterial adhesion to A549 cells.

We studied the transcriptional regulation mechanism of the *KP1_4563* gene by CRP. The results indicated that CRP negatively regulates *KP1_4563* by directly binding to the promoter region of *KP1_4563* and that *KP1_4563* negatively regulates the function of type 3 fimbriae in an unknown manner. Overall, CRP may indirectly and positively regulate the function of type 3 fimbriae. These results corroborate the importance of CRP in regulating virulence-related genes in *K*. *pneumoniae* [41].

CRP has been reported to be required for fimbrial production, and the deletion of *crp* lead to a huge attenuation of the ability to agglutinate yeast cells [61]. Our results clarify that CRP may indirectly and positively regulate the function of type 3 fimbriae by directly regulating the *KP1_4563* gene at the molecular level, which might aid in understanding the relationship of CRP and fimbriae. Lin reported that CRP down-regulates type 3 fimbriae expression indirectly through the c-di-GMP signaling pathway [62]. c-di-GMP is a second messenger molecule in bacteria, and the synthesis and decomposition of c-di-GMP is accomplished by diguanylate cyclases with conserved GGDEF domains and phosphodiesterases (PDEs) with EAL domains [63]. The *mrkJ* gene is immediately adjacent to the *mrkABCDF* operon that encodes the structural and assembly components of type 3 fimbriae. MrkJ has homology to EAL domain-containing phosphodiesterases (PDEs). Overexpression of *mrkJ* results in a significant decrease in the intracellular concentration of c-di-GMP and down-regulates type 3 fimbriae expression [64]. Bioinformatic analysis showed that the KP1_4563 protein does not contain conserved GGDEF or EAL domains, which indicates that *KP1_4563* negatively regulates the function of type 3 fimbriae without affecting the intracellular concentration of c-di-GMP, but did show that KP1_4563 protein has the putative conserved DUF1471 domains. In *E*. *coli* K-12, the YcfR protein has DUF1471 domains, and deleting *ycfR* caused changes in bacterial cell surface structures and properties by affecting cell surface protein gene expression, which further affects cell aggregation. The remarkable changes in the *ycfR* mutant may be due to regulation by CRP, as EMSA results showed that CRP binds to the upstream region of the *ycfR* gene [65]. We suspect that *KP1_4563* regulates the function of type 3 fimbriae, which may be associated with changes in bacterial cell surface structures and properties. Further study is needed to elucidate the precise role of *KP1_4563* in type 3 fimbrial function.

In conclusion, we found that *KP1_4563* negatively regulates type 3 fimbrial function, but does not influence type 1 fimbrial function. Adherence to A549 cells is considerably enhanced in the absence of *KP1_4563*. Moreover, CRP negatively regulates the transcription of *KP1_4563* by directly binding to the upstream *KP1_4563* promoter region.
